# Novel Cathelicidins from Pigeon Highlights Evolutionary Convergence in Avain Cathelicidins and Functions in Modulation of Innate Immunity

**DOI:** 10.1038/srep11082

**Published:** 2015-07-21

**Authors:** Haining Yu, Yiling Lu, Xue Qiao, Lin Wei, Tingting Fu, Shasha Cai, Chen Wang, Xuelian Liu, Shijun Zhong, Yipeng Wang

**Affiliations:** 1 School of Life Science and Biotechnology, Dalian University of Technology, Dalian, Liaoning 116024, China; 2 College of Pharmaceutical Sciences, Soochow University, Suzhou, Jiangsu, 215123 China

## Abstract

Cathelicidins are short cationic host defense peptides and play a central role in host innate immune system. Here we identified two novel cathelicidins, Cl-CATH2 and 3, from *Columba livia*. Evolutionary analysis of avian cathelicidins via phylogenetic tree and *Ka/Ks* calculations supported the positive selection that prompted evolution of CATH2 to CATH1 and 3, which originate from common ancestor and could belong to one superfamily. Cl-CATH2 and 3 both adopt amphipathic α-helical comformations identified by circular dichroism and the 3D structures built by Rosetta. Cl-CATH2 of CATH2 family with the most expression abundance in bird, exhibited relatively weak antimicrobial activity, but acted instead on the innate immune response without showing undesirable toxicities. In macrophages primed by LPS, Cl-CATH2 significantly down-regulated the gene and protein expressions of inducible nitric oxide synthase and pro-inflammatory cytokines while enhancing the anti-inflammatory cytokine, acting through MAPK and NF-κB signaling pathways. Molecular docking shows for the first time that cathelicidin binds to the opening region of LPS-binding pocket on myeloid differentiation factor 2 (MD-2) of toll-like receptor (TLR)4-MD-2 complex, which in turn inhibits the TLR4 pathway. Our results, therefore, provide new insight into the mechanism underlying the blockade of TLR4 signaling by cathelicidins.

Cathelicidins are a family of cationic peptides that play critical roles in the innate immune system^1^,^2^. They are most abundantly present in skin keratinocytes, mucosal epithelial cells, circulating neutrophils and myeloid bone marrow cells^3^,^4^. Cathelicidins generally exhibit unique bipartite features characterized by a substantial heterogeneous C-terminal mature peptide with remarkable structural diversity, which is linked to an evolutionarily conserved signal peptide and cathelin domain^3^,^4^,^5^. So far, cathelicidins have been identified from almost all vertebrate species, including mammals, birds, reptiles, amphibians and fishes^6^,^7^,^8^,^9^,^10^,^11^, with the hagfish cathelicidins as the most ancient members^12^. A gradual evolution model has been proposed, depicting the evolution pattern of cathelicidins from ancestral cystatin scaffold to current cathelin superfamily^13^. Conserved gene structure and related function provide evidence in favor of such a common ancestral origin for cathelicidins. Remarkable diversity in the mature peptide domain is a consequence of gene duplication followed by subsequent rapid divergence^1^,^14^,^15^. Unlike the highly distinct mammalian cathelicidins, the noticeable similarities of avian cathelicidins were observed even among different orders^16^. The mechanism underlying such evolution pattern of avian cathelicidin genes still remained obscure, and further calculation and evidence would be needed to have it fully stated.

Recently, emerging evidence suggests that cathelicidins as a member of natural host defense peptides may trigger a series of immunomodulatory responses in response to pathogen infection^17^,^18^. For mammalian cathelicidins, human LL-37 was proved to modulate immunity during bacterial infections by inducing IL-18 secretion through p38 and ERK1/2 MAPK activation in primary human keratinocytes^19^. SMAP-29 from sheep, CRAMP from mice and BMAP-28 from cattle were demonstrated to suppress the pro-inflammatory cytokine TNF secretion induced by microbial stimuli and increase the production of IgG1 in response to a Th2 biased environment (LPS and IL-4)^20^. OH-CATH30 from reptile, the king cobra, selectively up-regulates certain chemokines and cytokines production to protect mouse against sepsis^21^. Cathelicidin-PY from amphibian exhibited dual activities of antimicrobial and anti-inflammatory^22^. Likewise, asCATH1 and 2 from atlantic salmon fish reportedly stimulated the expression of the IL-8 in peripheral blood leukocytes^23^. However, for the aves, a niche bridging the evolutionary gap between reptilia and mammalian, there is few evidence of immunomodulatory role that cathelicidin plays in birds immunity, neither is the signaling pathway.

In the present work, we report a systematic characterization of two novel cathelicidins, Cl-CATH2 and 3 from the pigeon, *Columba livia* of Columbiformes (Aves) by molecular cloning, functional and structural studies. For the first time, the evolutionary relation of avian cathelicidins was interpreted from a new perspective, and furthermore, their families were re-classified based on the evolutionary convergence. Our work also provides new insights into the role that cathelicidin performs in the avian immunity. Cl-CATH2 enhanced the levels of anti–inflammatory cytokines such as IL-10, whilst suppressed the levels of LPS-stimulated pro-inflammatory cytokines including TNF-α, IL-6 and IL-1β. Thus, Cl-CATH2 was able to defense host against infections by modulating its immune response in addition to direct killing invading pathogens. Moreover, we also clarified signaling pathways involved in Cl-CATH2′s modulatory effect, and for the first time proposed the mechanism whereby Cl-CATH2 blocks TLR4 activation by LPS.

## Methods

### Collections of tissues

Two adult pigeons were purchased from local market. Tissue samples were collected from organs of spleen, bursa of fabrieius, heart, liver and lung according to standardised procedures. These tissues were dissected immediately and frozen in liquid nitrogen until use. All experiments were performed in accordance with relevant guidelines and regulations. All animal experiments are approved by Animal Care and Use Committee of Dalian University of Technology.

### SMART cDNA synthesis and molecular cloning of cathelicidins (Cl-CATHs)

The AxyPrepTM Multisource Total RNA Miniprep Kit (Invitrogen, USA) was used to extract total RNA from the bursa of fabrieius. In-FusionR SMARTer^TM^ PCR cDNA synthesis Kit (Clontech, Palo Alto, CA, USA) was used to prepare cDNA. The first-strand cDNA was synthesized using PowerScript Reverse Transcriptase by SMARTTM IV Oligonucleotide Primer 5′-AAGCAGTGGTATCAACGCAGAGTGGCCATTACGGCCGGG-3′ and CDS III/3′ PCR Primer 5′-ATTCTAGAGGCCGAGGCGGCCGACATGT(30)N−1N-3′(N = A, G, C or T; N−1 = A, G or C). The second strand was amplified using Advantage DNA Polymerase from Clontech by 5′ PCR primer 5′-AAGCAGTGGTATCAACGC AGAGT-3′ and CDS III⁄3′PCR primer.

A PCR-based method was used for screening the cathelicidin clones from the cDNA library. Two sense primers P1 (5′-ATGGCGAGCTGCTGGGCTGCT-3′) and P2 (5′-AACGCCTTCCAGGCTGCTCAG-3′) were designed from 5′-UTR and a highly conserved signal peptide domain, and coupled with CDS III/ 3′ PCR primer. The half nested PCR conditions involved two sections. 1st section using P1 combined with 3′ PCR primer was: 94 °C for 5 min; 26 cycles of 94 °C for 30 s, 57.9 °C for 30 s, 72 °C for 36 s; and again followed by a final extension at 72 °C for 10 min. 2nd section using the P2 combined with 3′ PCR primer was: 94 °C for 5 min; 26 cycles of 94 °C for 30 s, 56.8 °C for 30 s, 72 °C for 30 s; and followed by a 7 min extension period at 72 °C. DNA sequencing was performed on an Applied Biosystems DNA sequencer, model ABI PRISM 377.

### Expression profile by RT-PCR

Total RNA was extracted from different fresh tissues of pigeon. The first-strand cDNA was synthesized as described above, using forward primer P2 (5′-ACTTCACCATGATGGAGACCGAGTGC-3′) and reverse primer R2 (5′-ATTCTGCCCAGGAAGCGCCCGAACC-3′) for testing Cl-CATH2. PCR conditions were: 94 °C (1 min), and 30 cycles of 94 °C (30 s), 56 °C (30 s), 72 °C (1 min) followed by a 5 min extension period at 72 °C. The primers for testing Cl-CATH3 were: forward primer P3 (5′-CTGAGCCTGAAGGCGTTAATCT-3′) and reverse primer R3 (5′-GCATCAACCTCTACAAGGCCATCAAG-3′). The control PCR primers were 5′-actin (5′-ACACCCTACCTTGCTCACAGAA-3′, sense) and 3′-actin (5′-TGAAGAGTTCAGGAAGGGTTGA-3′, antisense).

### Phylogenetic tree construction and avian cathelicidins evolutionary analysis

Multiple avian cathelicidins were obtained from the protein database at the National Center for Biotechnology Information (NCBI). Multi-sequence alignments were constructed using clustalw, version 1.81 (http://www.ebi.ac.uk/clustalw/), based on the whole open reading frame (ORF). The phylogenetic trees were constructed by Neighbor-joining method (mega, version 5.0). Pairwise sequence alignment was performed specially for avian CATH2 family and galliformes (chicken) cathelicidins, respectively, using the Vector NTI software. *Ka/Ks* Calculator software package (version 1.2) was then exploited to estimate the nonsynonymos (*Ka*) and synonymous (*Ks*) substitution rates through model selection and model averaging.

### Peptide synthesis

Since cathelicidin of CATH2 family is ubiquitously found in all bird species studied so far, plus Cl-CATH3 is markedly conserved with Cc-CATH3 from quail, which has already been studied in our previous work^16^, only Cl-CATH2 was synthesized by GL Biochem Ltd. (Shanghai, China), and analyzed by HPLC and ESI-MS to confirm the purity higher than 95%.

### Antimicrobial and membrane permeabilization assay

More than 40 strains including standard and clinically isolated drug-resistant strains of bacteria and fungi were evaluated in the antimicrobial assay, which was conducted according to our previous methods^16^,^24^. The membrane permeabilization effect of Cl-CATH2 was investigated using the DNA-binding fluorescent dye propidium iodide (PI) according to Gao *et al*^25^. Briefly, bacterial strains were incubated in LB to OD600 of 0.5. Then Cl-CATH2 was added to 100 μl culture to make final concentration of 5xMIC. After 1 h incubation at 25 °C, the culture was centrifuged, resuspended in PBS, and then PI was added to a final concentration of 10 μg/ml. After 30 min incubation at 25 °C, the cells were washed 3 times with PBS and immediately photographed using fluorescence microscope (Olympus, Japan).

### Hemolytic and cytotoxic assays

Hemolytic activity on human erythrocytes was examined as previously described^26^, with 1% Triton X-100 as positive control. *In vitro* cytotoxicity against RAW264.7 murine macrophage cells was determined by MTT method^16^. All experiments were repeated three times.

### qRT-PCR

Murine macrophage Raw264.7 cells (3 × 10^5^/well) were treated with series concentrations of Cl-CATH2 and LPS (100 ng/ml) for 6 h. Total RNA was isolated using RNAiso Plus kit (Takara) and synthesized into cDNA with PrimeScript Reverse Transcriptase (Takara). The cDNA was assessed by qRT-PCR using SYBR green master mix kit (Takara) on a Realplex Mastercycler (Eppendorf, Germany). Gene expression was calculated after being normalized to those of GAPDH levels by ΔΔCt method^27^. The accuracy of the amplification reactions was achieved by melt curve analysis. The primers used are listed in Supplementary Table S1 online.

### NO and cytokines determination

RAW 264.7 cells (1 × 10^6^/ml) were cultured in DMEM (Gibco, Gaithersburg, USA) supplemented with 10% fetal bovine serum, 100 U/ml penicillin and 100 U/ml streptomycin in a humidified 5% CO_2_ atmosphere at 37 °C, and then plated and adhered to a 24-well culture plate (1 ml/well). The cells were incubated with 0 or 100 ng/ml LPS (from *E. coli* 055:B5, Sigma-Aldrich, USA) and serial concentrations of Cl-CATH2 (0, 5, 10 and 20 μg/ml) for 24 h. The culture medium was collected, and the level of NO was detected using Griess reagent (Beyotime, Jiangsu, China) by measuring absorbance at 540 nm on a microplate reader (Epoch Etock, BioTek, USA). The concentration of NO was deduced from the standard curve generated with control, NaNO_2_. The levels of inflammatory cytokines, including TNF-α, IL-6, IL-1β and IL-10, were examined in the cell culture supernatant using ELISA kits (Joyee, shanghai, china) according to the manufacturer’s instructions. The absorbance at 450 nm was measured on a microplate reader (Thermo Fisher, Langenselbold, Germany).

### Western blotting

RAW 264.7 cells (1 × 10^6^/ml) were cultured in serum-free DMEM for 16 h. The cells were incubated with Cl-CATH2 (0, 5, 10, and 20 μg/mL) for 1 h before the addition of LPS (0, 100 ng/mL). Three hours later, the cells were collected by centrifugation (1000 g for 5 min) and washed twice with ice-cold phosphate-buffered saline (PBS). The cytoplasmic proteins were then extracted from washed cell pellets using the Nuclear and Cytoplasmic Protein Extraction Kit (Beyotime, Jiangsu, China). Protein samples were separated by 10% SDS−PAGE and transferred to polyvinylidene difluoride (PVDF) membranes (Millipore, Bedford, MA). The membranes were blocked with 5% BSA and probed with the following specific primary antibodies against: GAPDH (1:3000, Beijing ComWin Biotech Co, China), p-ERK1/2, ERK1/2, p-JNK, JNK, p-p38, p38 and IκBα (1:2000; Cell Signaling Technology, Beverly, MA, USA). Blots were then incubated with HRP-conjugated goat anti-rabbit IgG (Cell Signaling Technology) and developed using the SuperSignal WestPico chemiluminescence substrate (Pierce Chemical Co.).

### Structural characterization

CD spectroscopy was performed using a Jasco J-715 spectrophotometer. Peptide (0.5 mg/ml) was prepared in water and 50% (v⁄v) trifluoroethanol (TFE)⁄water, respectively, and then added into a quartz optical cell with a path length of 0.5 mm at 25 °C. The spectra were averaged over three consecutive scans, followed by subtraction of the CD signal of the solvent. The secondary structure content was estimated by PSIPRED v3.3, and the amphipathicity of the peptide was estimated by plotting the helical wheel diagrams (http://heliquest.ipmc.cnrs.fr/cgi-bin/ComputParamsV2.py).

The 3D structures of Cl-CATH2 and 3 were manually built using Rosetta *ab initio* software (version 3.5)^28^. Totally 5570 and 5000 decoys were generated for Cl-CATH2 and 3, respectively, among which the structure with lowest energy were further verified by PROCHECK^29^. Then the comparative 3D structure models of Cl-CATH2 and 3 were visualized using PYMOL software (http://www.pymol.org) without any other refinements.

### Molecular docking

The constructed 3D structure of Cl-CATH2 was next subject to molecular docking. The crystallographic structure of TLR4-MD-2 complex was obtained from PDB bank (PDB code: 2Z64), and visualized using PYMOL. The initial complex structure of TLR4-MD-2-Cl-CATH2 was accomplished by Zdock3.0.2. Totally 3600 decoy structures through the Zdock rigid-binding were predicted, and among them, the decoy with the lowest energy was chosen for the following flexible docking study. 10000 decoy structures were obtained from the flexible docking by RosettaDock (version3.5), among which the plausible docking model with the lowest interface binding energy was selected for further analysis.

## Results

### Identification and characterization of pigeon cathelicidins

**Figure 1:**
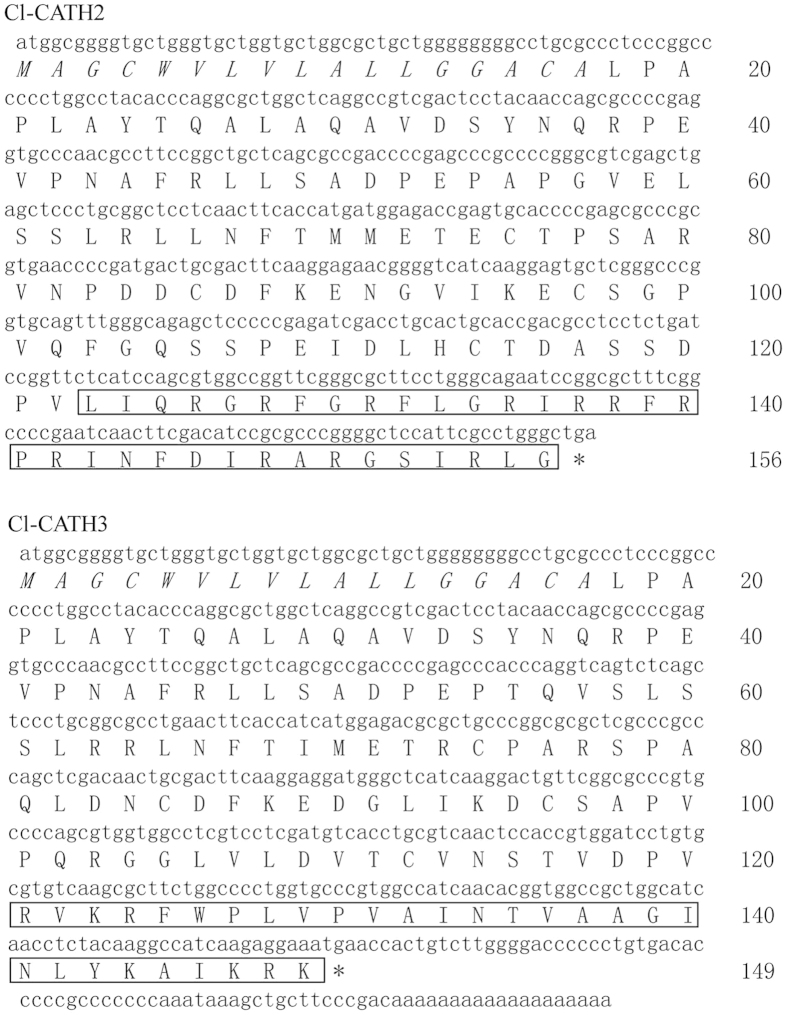
The cDNA sequence encoding Cl-CATH2 and Cl-CATH3 (*Columba livia*) as well as the deduced precursor amino acid sequence. The predicted signal peptides of Cl-CATHs are shown in italic and deduced mature peptides of Cl-CATHs are boxed. (^*^) indicates the stop codon.

From the cDNA library constructed, two clones encoding two novel cathelicidins were isolated and sequenced. The deduced 156-aa and 149-aa precursors both contain a 17-aa N-terminal signal domain predicted by signalp 3.0 (Fig. 1). Sequence alignments showed that avian cathelicidins all share same gene structure: a signal peptide followed by a conserved cathelin domain with four invariantly spaced cysteines and a cationic C-terminal mature antimicrobial peptide (see Supplementary Fig. S1 online). Two mature peptides were predicted, Cl-CATH2 (LIQRGRFGRFLGRIRRFRPRINFDIRARGSIRLG, 34aa) (GenBank Access number: KP645199) and Cl-CATH3 (RVKRFWPLVPVAINTVAAGINLYKAIKRK, 29aa) (GenBank Access number: KP645200). The theoretical pI/Mw predicted using Protparam tool (http://au.expasy.org/tools/protparam.html) for Cl-CATH2 and 3 are 12.65/4127.92 and 11.75/3323.08, respectively.

Expression profile evaluated by semi-quantitative RT-PCR indicated a broad tissue distribution for both cathelicidins except muscle, with a slightly different pattern (see Supplementary Fig. S2 online). The expression of Cl-CATH2 was more abundant than Cl-CATH3 in spleen, lung, bursa and liver, whilst in kidney and heart, Cl-CATH3 was one with higher expression.

### Phylogenetic and evolutionary analyses

**Figure 2:**
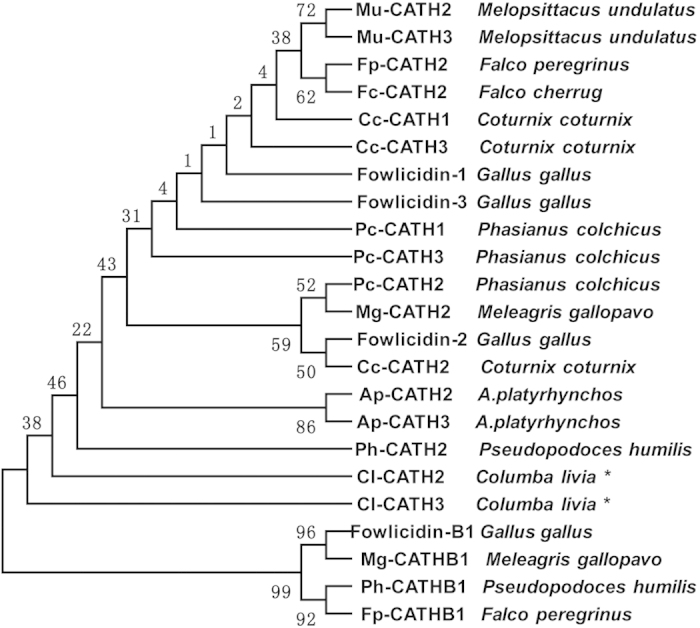
Phylogenetic analyses of Cl-CATH2, Cl-CATH3 and all avian cathelicidins on the basis of the proregions. The phylogenetic dendrogram was constructed by the Neighbor-joining method based on the proportion difference of aligned amino acid sites of the sequence. Only branches supported by a bootstrap value (expressed as percentage of 1000 bootstrap samples supporting the branch) are shown at the branching points.

The multi avian cathelicidins alignment was performed on basis of the precursors. A condensed multifurcating tree was constructed, emphasizing the reliable portion of pattern branches (Fig. 2). It is demonstrated that all avian cathelicidins form two distinct clusters, with B1 family located in a separated clade from others. Families of CATH1, 2 and 3 are in the second, also the main cluster, and undoubtedly show evolutionary ‘closeness’ to each other, suggesting that CATH1~3 families arose from a common ancestor. To further probe the evolutional relationships among avian cathelicidin families, the ratio of the number of nonsynonymous substitutions per non-synonymous site (*Ka*) to the number of synonymous substitutions per synonymous site (*Ks*), the *Ka/Ks* ratio, was calculated for each gene domain of chicken cathelicidins individually (see Supplementary Table S2 online). The results show that B1 undoubtedly represents the oldest gene family among all, and positive selection has greatly prompted evolution of cathelicidin sequence to smaller size and better activity. Besides, it is also indicated that CATH2 has a longer evolutionary history than CATH1 and 3, which is further approved by the positive selection targeting the mature peptide domain driven by pressures of pathogen diversity and virulence, since there was a tendency that CATH1s and 3s are evidently more efficacious than CATH-2s in killing pathogens^16^,^24^,^30^.

The *Ka/Ks* ratio was also computed for CATH2 family alone (see Supplementary Table S3 online). Signal peptide region is absent of synonymous substitutions (*Ks* = 0), demonstrating that positive selection could have driven the evolution of this region more favorable than the other two. For the region of mature peptide, most pairwise *Ka/Ks* ratios are less than 1, suggestive of the negative selection, in which case, most residue changes are deleterious and, thereby, are selected against. So far, the reason why cathelicidins from different orders (Aves) literally display such significant similarities in each genetic domain still remained unpuzzled. Further investigation on the functional role of C-terminal domain will be critical to interpret these observations.

### Solution structures

**Figure 3:**
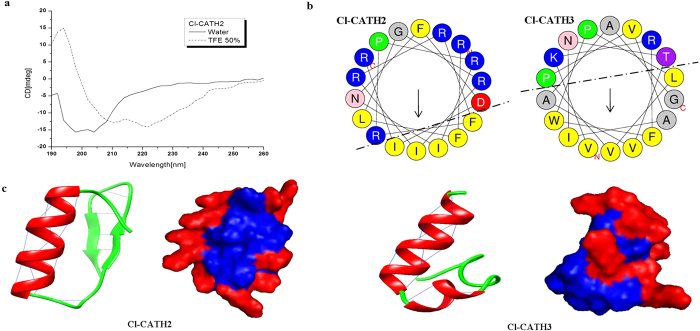
(**a**) Circular dichroism analysis of Cl-CATH2 in different solvent environments. (**b**) helix-wheel plots and (**c**) tertiary structures built by Rosetta *ab initio* of Cl-CATH2 and Cl-CATH3. For helix-wheel plots, the hydrophobic and hydrophilic residues are separated with dash dot line, with the hydrophilic residues being concentrated on upper side of the helix and hydrophobic ones on the lower. For tertiary structures, intramolecular hydrogen bonds and α-helix are displayed in the ribbon structure. Surface representation of Cl-CATH2 and 3 are shown in blue with positive charges shown in red region.

The CD spectrum of Cl-CATH2 in the membrane-like environment of 50% TFE/H2O showed one positive band (190 nm) and two negative dichroic bands at 208 and 222 nm, consistent with the α-helical conformations (Fig. 3a). This is concordant with prediction of PSIPRED (v3.3) that generates a 26.47% and 68.97% α-helical component for Cl-CATH2 and 3, respectively. Helical wheel analysis shows that sequences derived from Cl-CATH2 (residues 9–26), Cl-CATH3 (residues 2–19) both form distinct amphipathic structures, and Cl-CATH3 shows more amphipathic than Cl-CATH2 (Fig. 3b), with hydrophilic residues facing upward, while hydrophobic ones facing downward. For Cl-CATH2 and 3, 5 residues (FFIII) and 11 residues (LGAFVVVIWAP) constitute the hydrophobic face, respectively, whereas the other residues comprise the hydrophilic section. Such amphipathic alpha-helix structure is usually adopted by most of small cationic peptides and is thought to be important for their disrupting the membrane integrity^31^,^32^. The tertiary structures of Cl-CATH2 and 3 built by Rosetta *ab initio* also demonstrated the components of α-helix and its relative content, highly consistent with the secondary structure predictions (Fig. 3c). Besides, the antiparallel β-strands was also observed juxtaposed the helix in the Cl-CATH2. Cl-CATH3 was also shown to adopt an α-helical structure that contains two antiparallel α-helixes. The positive-charged residues are distributed along the molecular surface to make Cl-CATH2 electrostatic and capable to bind the negative-charged bacterial surface and LPS (Fig. 3c).

### Antimicrobial activity and action mode

**Figure 4:**
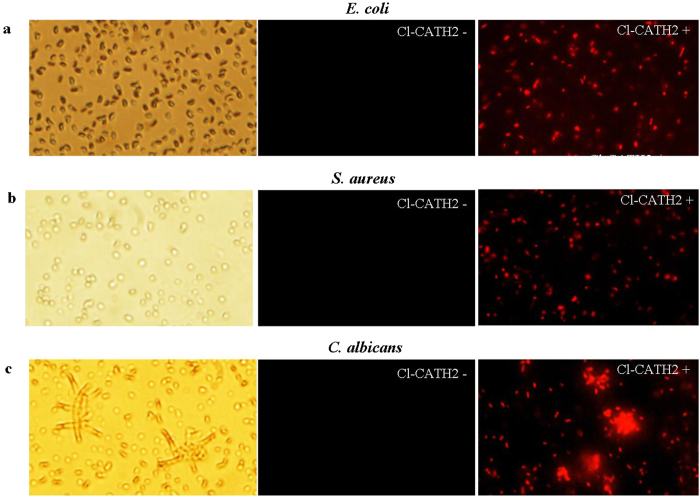
Membrane permeabilization of Cl-CATH2 on *E. coli* (**a**), *S. aureus* (**b**) and *C. albicans* (**c**). The left and middle columns represent the microorganisms in absence (−) of Cl-CATH2, under visible light and fluorescence, respectively; the right column is the microorganisms in presence (+) of Cl-CATH2 under fluorescence.

Cl-CATH2 exerted broad-spectrum but moderate antimicrobial abilities with most MICs ranging from 9.375 to 37.5 μg/ml (Table 1). Although both clinically isolated drug-resistant and standard strains are susceptible to Cl-CATH2, MIC values appear much higher compared to Cc-CATH3^16^, fowlicidin-1^30^ and Pc-CATH1^24^. To determine the possible mechanism underlying Cl-CATH2′s antimicrobial action, its membrane permeabilization effect on *E. coli*, *S. aureus* and *C. albicans* were tested using the classical DNA-binding dye PI. As shown in Fig. 4, the red dye that stains only dead cells with broken membranes was significantly increased, suggesting that the membranes of microorganisms have been disrupted by Cl-CATH2 in a mode similar to that of other amphipathic alpha-helix AMPs.

### Hemolysis and cytotoxicity

The hemolytic and cytotoxic activities of Cl-CATH2 were examined using the fresh-prepared human erythrocytes and RAW 264.7 macrophage cells, respectively. At a concentration up to 200 μg/ml, Cl-CATH2 only induced 12.84% hemolysis. At a concentration of 50, 100, and 200 μg/mL, Cl-CATH2 could induce 2.84, 4.72 and 13.27% death of RAW 264.7 cells, respectively.

### Immunomodulatory activities of Cl-CATH2

**Figure 5:**
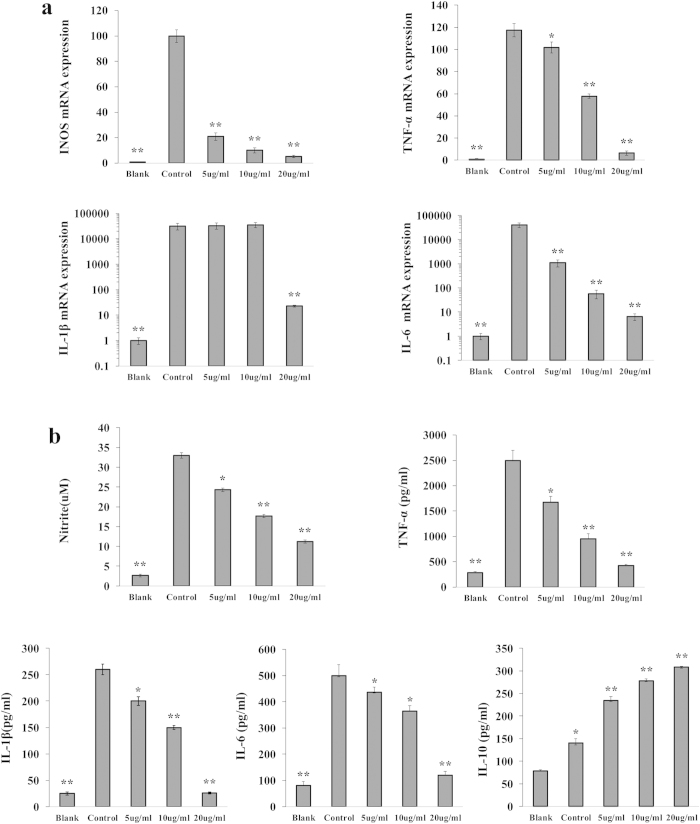
(**a**) Effects of Cl-CATH2 on iNOS and pro-inflammatory cytokine expression induced by LPS. Expression of the target genes iNOS, TNF-α, IL-1β, IL-6 and GAPDH was measured by real-time PCR and presented as fold change in the target genes expression normalized to GAPDH. Fold change of target genes expression in untreated cells was normalized to 1. Data are presented as the mean ± SD from three independent experiments (^*^, P < 0.05; ^**^, P < 0.01; by unpaired t test). (**b**) Effects of Cl-CATH2 on NO and cytokine secretions stimulated by LPS. NO, TNF-α, IL-1β, IL-6 and IL-10 in RAW264.7 cell culture supernatant was determined by Griess reagent and ELISA, respectively. Blank: without LPS and Cl-CATH2. Control: cells only treated with LPS. Data are presented as the mean ± SD from three independent experiments (^*^, P < 0.05; ^**^, P < 0.01; by unpaired t test).

To further determine whether Cl-CATH2 can confer protection against infection by triggering an immune response other than simply killing microbes, the cytokine productions were evaluated in macrophage cells after treatment with LPS and different concentrations of Cl-CATH2 using qRT-PCR. mRNA levels of the INOS (inducible nitric oxide synthase) and couple of key pro-inflammatory cytokines, such as TNF-α, IL-1β, and IL-6 were all significantly upregulated after stimulation with LPS alone (Fig. 5a). However, expressions of all cytokines examined except IL-1β were markedly suppressed by Cl-CATH2 in a dose-dependent fashion, and that of IL-1β was not obviously altered until the concentration of Cl-CATH2 up to 20 μg/ml (Fig. 5a). The mRNA level of INOS was most sensitive to Cl-CATH2′s modulation. At Cl-CATH2 concentrations of 5, 10 and 20 μg/ml, the expression level of INOS was inhibited by 79%, 90% and 95%, respectively (Fig. 5a). Cl-CATH2 at 4.85 μM (20 μg/ml) significantly suppressed the INOS, TNF-α, IL-1β and IL-6 mRNA levels by 94.8%, 94.5%, 99.93% and 99.98%, respectively, which is much more potent than other cathelicidins reported so far., SMAP-29 (20 μM) failed to downregulate the expressions of IL-1β and CCL-2/MCP-1^20^, albeit possessing immunomodulatory activity. Chicken fowlicidin1/2 (20 μM) only decreased the same gene levels by 90%^30^. asCATHs from salmon was found no regulating activities upon the expression of IL-1 or IL-18^23^.

It is also confirmed by ELISA that LPS-induced protein levels of TNF-α, IL-1β and IL-6, and NO level were also remarkably and dose-dependently blocked in macrophage cells by Cl-CATH2 (Fig. 5b). At Cl-CATH2′s concentration of 4.85 μM (20 μg/ml), they were inhibited by 83.2%, 90%, 76% and 66.05%, respectively. In contrast, the level of anti-inflammatory cytokine, IL-10 was raised by nearly 2-fold after LPS’s stimulation alone, and was more incremented upon Cl-CATH2 treatment together (Fig. 5b). OH-CATH30 from the king cobra is one of a few cathelicidins that have been studied for regulating both pro- and anti-inflammatory cytokines^21^. The concentration of IL-10 induced by Cl-CATH2 and OH-CATH30 (both at 10 μg/ml) were 280 pg/ml and 156 pg/ml, respectively. Cathelicidin-PY (20 μg/m) from amphibian only inhibited LPS-induced TNF-α, IL-6, and MCP-1 by 60, 86, and 68%, respectively^22^. Collectively, these data strongly proved the ability of Cl-CATH2 to initiate a range of immunoregulatory responses in pigeon of Aves, enhancing the level of anti-inflammatory cytokine while reducing those of proinflammatory cytokines.

### Interaction of Cl-CATH2 and TLR4

**Figure 6:**
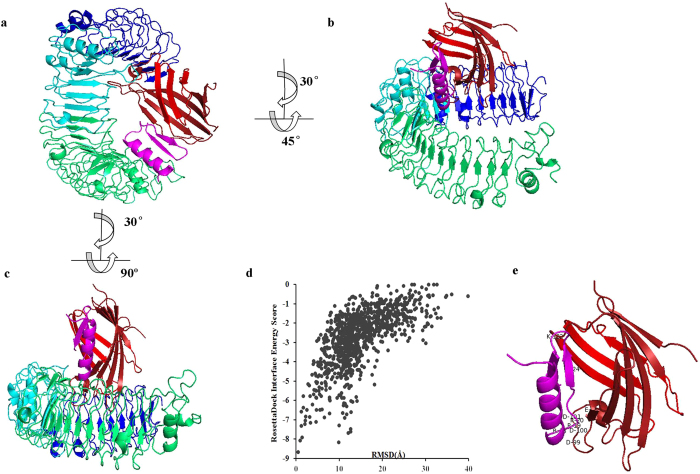
Overall structure of Cl-CATH2-TLR4-MD-2 Complex. (**a**–**c**) shows three views of Cl-CATH2 binding to mouse TLR4-MD-2 Complex. The N-terminal, central, and C-terminal domains of TLR4 are displayed in blue, cyan and green, respectively. The β-strands forming the MD-2 “cup” are colored in red and firebrick, and Cl-CATH2 is in magenta. (**d**) Energy versus rmsd plot. 1000 out of 10000 decoy structures from flexible docking study of TLR4-MD-2-Cl-CATH2 by RosettaDock. The horizontal axis represents the rmsd value compared to initial structure during flexible docking whereas the vertical axis represents the interface binding energy score of RosettaDock. (**e**) Closeup view of Cl-CATH2 binding to the mouse MD-2. Residues involved in interaction between Cl-CATH2 and MD-2 are displayed.

Innate immunity is triggered by the binding of LPS, also known as one of the pathogen-associated molecular patterns, with pattern-recognition receptors, including TLRs^33^. Shortly after that, a broad, nonspecific innate immune response takes place, and the innate immune cells is triggered to secrete a range of inflammatory effectors^33^,^34^. TLR4 was determined not to bind LPS alone, but form a TLR4-MD-2 complex with MD-2 protein that interacts with LPS^35^,^36^. Whereafter LPS causes dimerization of the TLR4-MD-2 complex and initiates intracellular signaling^37^. To probe how Cl-CATH2 blocks the LPS-stimulated TLR4 signaling, the binding mode of Cl-CATH2 to TLR4-MD-2 complex was analyzed by molecular docking^38^. Cl-CATH2 binds to the C-terminal domains of MD-2 and cross over the MD-2 pocket edge, which covers the binding pockets of LPS, and in turn blocks the LPS binding to MD-2 followed by inhibition of TLR4 signaling (Fig. 6a–d). The mechanism for this Cl-CATH2-TLR4-MD-2 conformation possibly lies in lacking efficient hydrophobic interactions between Cl-CATH2 and interior of MD-2 pocket that is inclined to bind molecules like LPS with multiple hydrophobic acyl chains^37^. The interaction with Cl-CATH2 and MD-2 was principally mediated by ionic interactions, between Asp24 (Cl-CATH2)-Lys125 (MD-2) and tandem of Arg18-Phe19-Arg20 (Cl-CATH2) and Asp99-Asp100-Asp101 (MD-2) (Fig. 6e).

### Signaling pathways involved in Cl-CATH2′s regulation of immune response

**Figure 7:**
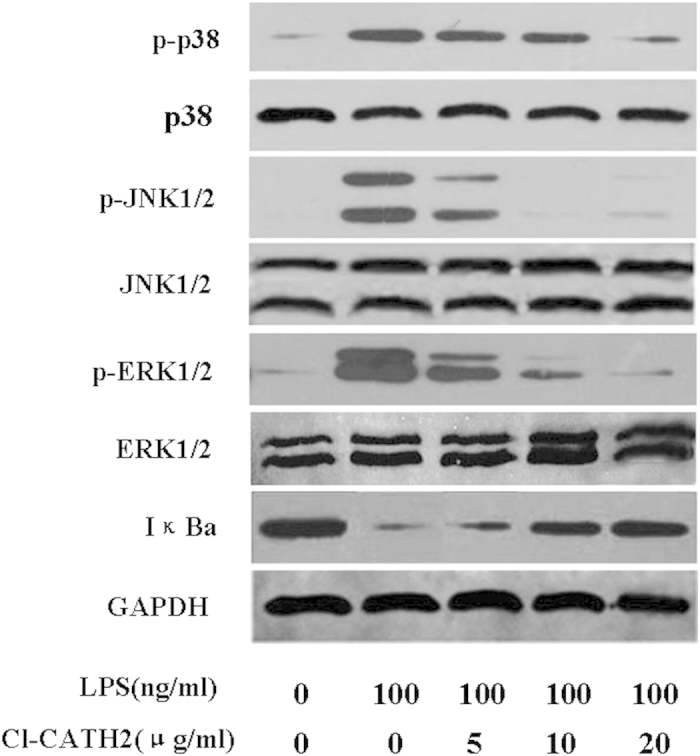
Effects of Cl-CATH2 on innate immune response signaling pathways. Raw 264.7 macrophage cells were treated with or without LPS and/or Cl-CATH2 of indicated concentration. Expression of specific proteins p38, JNK1/2, ERK1/2 and IκBα was tested by western-blotting.

Studies were undertaken to probe the mechanism by which Cl-CATH2 regulated the innate immune responses in bird. Once the LPS, one of the most potent immunostimulants, is recognized by TLR4 on assorted cells (macrophages, dendritic cells, B cells, specific types of T cells, fibroblasts and epithelial cells)^39^, the innate immune responses are triggered via the TLR signaling. Thereby, the effects of Cl-CATH2 on the TLR4-mediated immune response were examined. Results indicated that Cl-CATH2 substantially inhibited p38, ERK1/2 and JNK1/2 of the mitogen-activated protein kinase (MAPK) signaling in a concentration-dependent manner (Fig. 7). At Cl-CATH2′s concentration of 20 μg/ml, p-p38 and p-ERK1/2 were suppressed by 83% and 92%/82%, respectively. The p-JNK1/2 induced by LPS was almost completely blocked (98.2%/98.4%) by Cl-CATH2 of 20 μg/ml. To further investigate whether Cl-CATH2 ultimately blocks the activation of NF-κB, the effect of Cl-CATH2 on the IκBα degradation was determinated. IκB is a cytoplasmic protein complex that inactivates NF-κB. Cl-CATH2 of 5, 10 and 20 μg/ml inhibited the LPS-induced degradation of IκBα by 2.29, 2.77 and 2.53 fold, respectively (Fig. 7). Comparatively, LL37 (10 μg/ml) was reported to only slightly increase the phosphorylation of NF-κB, p65^21^.

## Discussion

Cathelicidins, firstly discovered in bovine neutrophils, are recognized as a young family of endogenous antibiotics, and perform a critical role in host innate immune defense against invasive bacterial infection^3^,^40^. The gene structures of cathelicidins from various vertebrate species seem quite conserved, whilst the mature peptides encoded are virtually much diversified, especially for the mammals. However, unlike the highly distinct mammalian cathelicidins resulting from repeating gene duplication events, cathelicidins of avian share significant similarity, except for B1 family. Even residue mutation in C-terminus is a rare occurrence for each avian cathelicidin family (CATH 1~3). Judging by the sequences alignment and phylogenetic analysis, CATH1 and 3 might originate from a common ancestor through gene duplication, which is not ubiquitous in all birds though, but only takes place in three species (*G. gallus*, *C. coturnix* and *P. colchicum*). Further evidence comes from their similar biological activity and potency^24^,^30^. By contrast, CATH2 is the most abundantly expressed cathelicidin gene in bird, following the CATH3. Despite CATH2s’ relatively low sequence similarity with CATH1s and CATH3s, *Ka/Ks* values between gene pairs, such as fowlicidin-1/fowlicidin-2, fowlicidin-2/fowlicidin-3, are still below 1, indicative of the negative selection. High conservation of avian cathelicidins could be due to the slowed amino acid substitution in the subsequent evolution for fighting less microbial pathogens in their much simpler habitat than mammals, reptiles, amphibians and fishes. The other possible explanation might be that the fairly strong activity of avian cathelicidins (compared with Reptilia and Mammalia) exempted them undergo too much gene evolutions.

In current work, the antimicrobial activity of Cl-CATH2 from avian CATH2 family was determined as broad-spectrum, but weaker than CATH1s and CATH3s^16^,^24^,^30^. Structural differences in amphipathic balance and alpha-helix content may account for their differential potency. Cl-CATH3 of CATH3 family clearly showed more hydrophobicity and helix compared to Cl-CATH2. Generally, hydrophobicity is tightly associated with loss of cytolytic activity^41^, which probably explains Cl-CATH2′s neglectable hemolytic and cytotoxic activities. The structure analysis also indicated that both Cl-CATH2 and 3 have structurally adjacent cationic residues (Lys and Arg) located at the exterior of the molecular, which facilitate the interaction with negatively charged cell surface. The antimicrobial action mode of Cl-CATH2 determined by membrane permeabilization assay was concordant with previously proposed barrel-stave model^42^, that is, perturbation of the cell membrane.

Inflammation is a mechanism for maintaining homoeostasis in response to detrimental stimuli like infection. Recently, it has been suggested that the host’s protective antimicrobial immunity can be enhanced by limiting the inflammation-induced tissue injury^33^. To understand the actual role that cathelicidin plays in the avian innate immune response to infection challenge, the ability of Cl-CATH2 to modulate the TLR-mediated inflammatory responses induced by LPS was investigated. LPS binding with the extracellular portion of TLR4-MD2 complex on macrophages can trigger the secretion of various inflammatory effectors that lead to severe infections^43^, which was similar with the cytokine profile induced by LPS in the present work. Here, Cl-CATH2 prominently blocked the genes of LPS–induced key pro-inflammatory cytokines (TNF-α, IL-6, and IL-1β) and iNOS, which can produce NO in a variety of tissues, and detectable in many cell types associated with infection and inflammation^44^. Moreover, studies at the protein level indicated the concordant cytokine and NO changes in supernatants of Cl-CATH2–treated macrophages. Nevertheless, Cl-CATH2 selectively increases the production of anti-inflammatory cytokine, IL-10. These results suggest that Cl-CATH2 can modulate the innate immune response by balancing or controlling instead of simply inhibiting the immune response during protection from infection.

In the TLR4-MD-2 complex, MD-2 is the LPS-binding component^36^. Once the TLR4/MD-2 complex recognizes LPS, the TLR signaling pathway is activated, and ultimately leads to the activation of multiple downstream molecules, such as MAPKs, NF-κB, and IFN-regulatory factors (IRFs), driving the subsequent production of pro-inflammatory cytokines, chemokines and type I interferons (IFNs) and, consequently, promoting the antimicrobial immunity^45^. TLRs are quickly expressed on various immune cells in response to pathogens, cytokines, and environmental stresses^37^. Previous studies have demonstrated that cathelicidins had inhibitory effects on the TLR4 expression induced by LPS, such as cathelicidin-PY from amphibian^22^. OH-CATH30 from reptile was also shown to down-regulate the TLR4/MD2 complex level by 20%^21^. These effects might result from the neutralization of LPS by cathelicidin. However, the action mode of cathelicidins upon TLR4 expressed extracellularly and mechanism underlying their interactions still remains unclear. The molecular docking result demonstrates for the first time that in addition to direct neutralization of LPS, cathelicidin also binds to the opening region of the MD-2 pocket with no direct interaction with TLR4, which in turn blocks LPS entering into the pocket and following TLR4 signaling pathway activition.

Despite the fact that cathelicidins in bird are immunomodulatory, none of them are studied for the mechanisms through which they exert effects. Here, the effect of Cl-CATH2 on MAPK, NF-κB signaling pathways was investigated using western blot. Cl-CATH2 significantly suppressed the JNK1/2 and ERK1/2 signaling pathways, but only blocked the p38 MAPK modestly. Besides, the inhibitory effect of Cl-CATH2 on NF-κB signaling pathway was not as potent as that on MAPK pathways, only ranging 2.29 to 2.77 fold under concentrations tested of Cl-CATH2. It indicates that Cl-CATH2 might selectively regulate the innate immune response via MAPK signaling pathways.

In summary, the identification and characterization of cathelicidins (Cl-CATH2/3) from *Columba livia* were reported in current study. Structural and phylogenetic analyses indicate that avian cathelicidins represent a good example for convergent evolution. The structural restraints especially between avian CATH1s and CATH3s might originate from their similar functional features. The avian CATH2 family has a 3 ~ 8-residue extension in their C terminus compared to CATH1s and CATH3s, leading to their functional divergence. As reflected by the functional assays, Cl-CATH2 was demonstrated to contribute to bird’s innate defense mainly by modulating the innate immune responses. Nowadays, such immunomodulatory properties of host defense peptides are of particular interest for development the novel anti-infection agent with no drug resistance, since the peptides mainly act on the host instead of pathogens. Besides, the Cl-CATH2′s blockade of pro-inflammatory cytokines makes it valuable in using as the adjuvants for vaccination or anti-inflammatory agent for treating acute and chronic inflammatory disorders.

## Additional Information

**How to cite this article**: Yu, H. *et al.* Novel Cathelicidins from Pigeon Highlights Evolutionary Convergence in Avain Cathelicidins and Functions in Modulation of Innate Immunity. *Sci. Rep.* **5**, 11082; doi: 10.1038/srep11082 (2015).

## Supplementary Material

Supplementary Information

## Figures and Tables

**Table 1:** Antimicrobial activities of Cl-CATH2.

**Microorganisms**	**MICª(ug/ml)**
**Gram-Negative Bacteria**	
*Escherichia coli* ATCC25922	18.75
*E. coli* 08040726 (CI, DR)	37.50
*Klebsiella Trevisan* 1400 (CI, DR)	18.75
*Pseudomonsa aeruginosa* ATCC27853	18.75
*Stenotrophomonas maltophilia* 7407(CI, DR)	75
**Gram-Positive Bacteria**	
*Staphylococcus aureus* ATCC27853	9.38
*S. aureus* 08032706 (CI, DR)	18.75
*S. aureus* 08032712 (CI, DR)	18.75
*S. aureus* 08032810 (CI, DR)	9.38
*S. aureus* gz130623 (CI, DR)	18.75
*Bacillus cereus* 1373(CI, DR)	9.38
*Bacillus subtilis* 1345 (CI, DR)	75
*Enterococcus faecalis* 1396 (CI, DR)	75
**Fungi**	
*Candida albicans* ATCC2002	37.50
*C. albicans* 08022821(CI, DR)	75
*C. albicans* 08030809(CI, DR)	75
*C. albicans* 08030102(CI, DR)	150
*Candida glabrata* 08A802(CI, DR)	18.75
*C. glabrata* 091223	150

MIC, minimal inhibitory concentration,these concentrations represent the mean values of three independent experiments performed in duplicate. CI: clinically isolated strain. DR: drug resistance for ampicillin and benzylpencillin.
