# Supplementary material for: Novel Cathelicidins from Pigeon Highlights Evolutionary Convergence in Avain Cathelicidins and Functions in Modulation of Innate Immunity
Source: Sci Rep. 2015 Jul 21;5:11082. doi: 10.1038/srep11082 (PMC4508531; doi:10.1038/srep11082)
Supplement: Supplementary Information [file srep11082-s1.pdf]

Novel Cathelicidins from Pigeon Highlights Evolutionary Convergence in Avain Cathelicidins and Functions in Modulation of Innate Immunity

Haining Yu[1]*, Yiling Lu[1¶], Lin Wei[2¶], Tingting Fu[1], Xue Qiao[1], Shasha Cai[1], Chen Wang[1], Xuelian Liu[1], Shijun Zhong[1], Yipeng Wang[2]*

From the [1]School of Life Science and Biotechnology, Dalian University of Technology, Dalian, Liaoning 116024, China;
[2]College of Pharmaceutical Sciences, Soochow University, Suzhou, Jiangsu, 215123 China

[¶]These authors have the same contribution to this paper
* Author to whom correspondence should be addressed:
[1]Haining Yu: Tel (Fax): 86-411-84708850; E-mail: joannyu@live.cn.
[2]Yipeng Wang: E-mail:yipengwang@suda.edu.cn

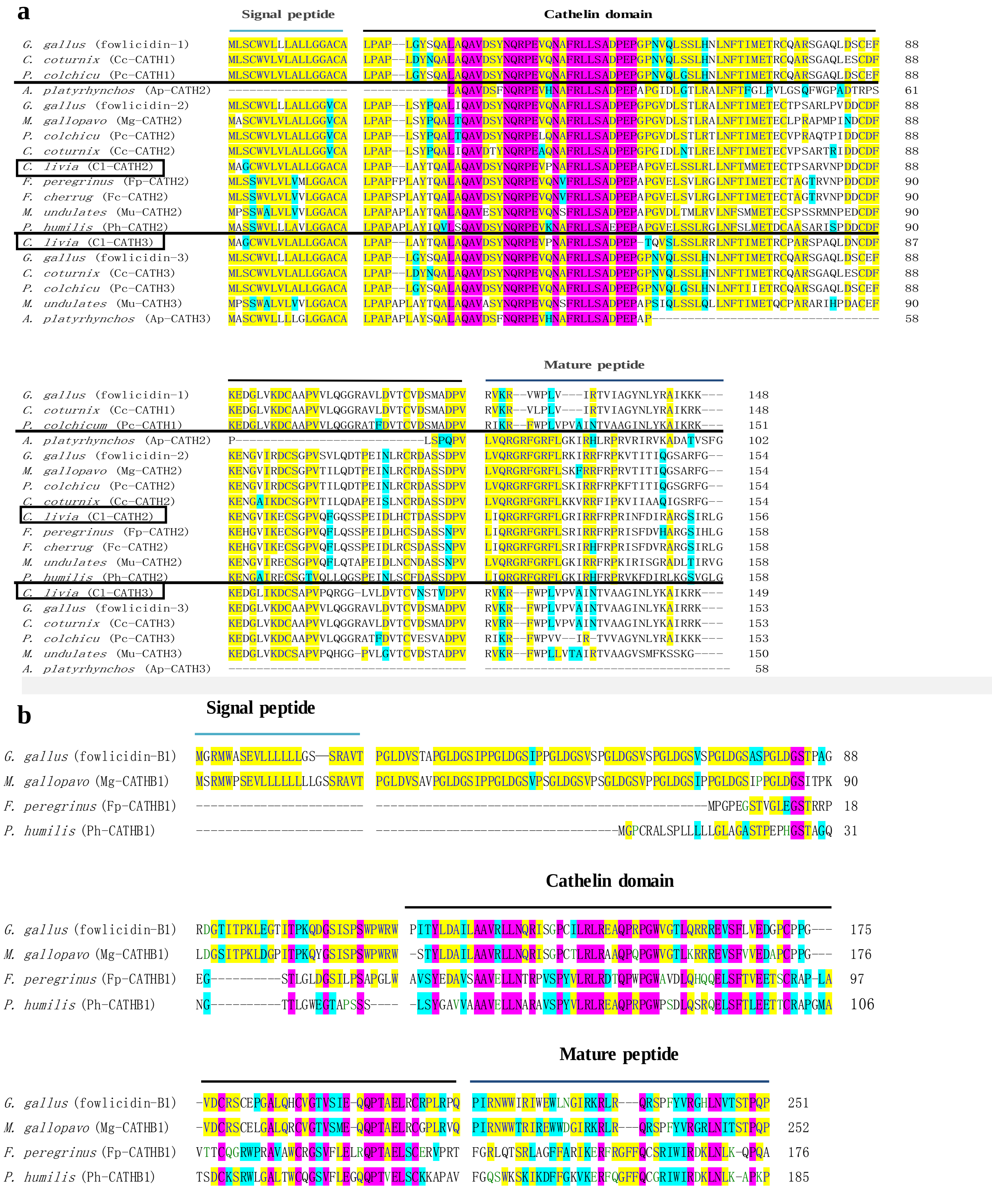

Figure S1 Multiple sequence alignment of various avian cathelicidin families. (a). CATH1~3; (b). CATH-B1. Dashes are inserted to optimize the alignment, and conserved residues are shaded. The domains of signal peptide, cathelin and mature peptide are labeled.

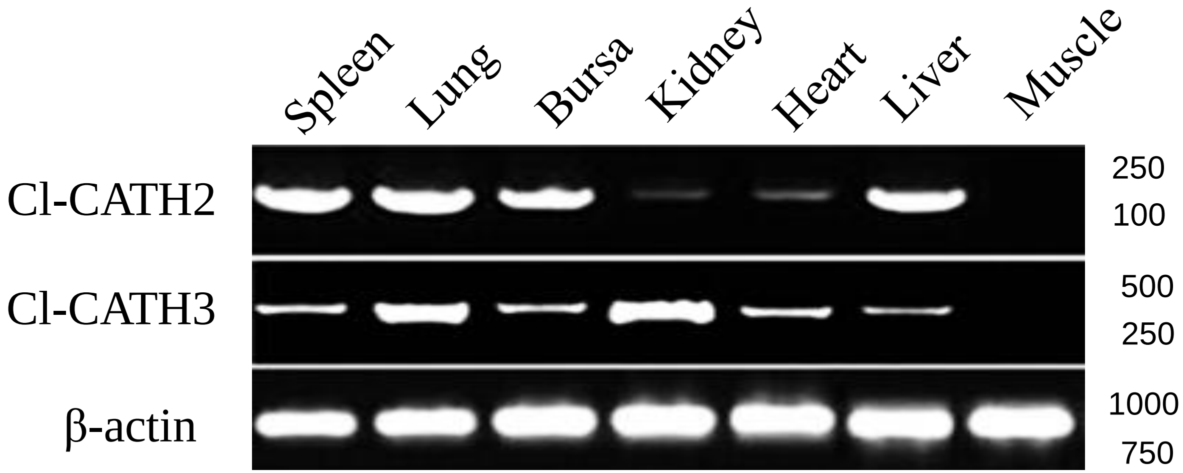

Figure S2 │ Expression profile of Cl-CATH2 and Cl-CATH3 using gene-specific primers. RNA normalization was confirmed by similar tissue distribution of β-actin housekeeping.

Table S1 │ The primers used for qRT-PCR analysis.

iNOS	Forward	5'-CTGCAGCACTTGGATCAGGAACCTG-3'
	Reverse	5'-GGAGTAGCCTGTGTGCACCTGGAA-3'
TNF-a	Forward	5'-CGGTGCCTATGTCTCAGCCT-3'
	Reverse	5'-GAGGGTCTGGGCCATAGAAC-3'
IL-1β	Forward	5'-ATGGCAACTGTTCCTGAACTC-3'
	Reverse	5'-GCCCATACTTTAGGAAGACA-3'
IL-6	Forward	5'-GAGGATACCACTCCCAACAGACC-3'
	Reverse	5'-AAGTGCATCATCGTTGTTCATACA-3'
GAPDH	Forward	5'-AAGCCCATCACCATCTTCCA-3'
	Reverse	5'-CCTGCTTCACCACCTTCTTG -3'

Table S2 │ Evidence for adaptive evolution different avian cathelicidin families, with fowlicidins as representatives. a, multiple sequence alignment of the precursors of fowlicidins from *G. gallus.* Idential residues are shaded. b, nonsynonymous substitution rate *Ka*, synonymous substitution rate *Ks*, and the ratio between them. NA , not applicable (*Ks* = 0).

a

```
                                     Signal peptide
G. gallus (fowlicidin-1)   (1)  ---MLSCWVLLLALLGGACA  --------------------------------------------------------------------
G. gallus (fowlicidin-2)   (1)  ---MLSCWVLLLALLGGVCA  --------------------------------------------------------------------
G. gallus (fowlicidin-3)   (1)  ---MLSCWVLLLALLGGACA  --------------------------------------------------------------------
G. gallus (fowlicidin-B1)  (1)  MGRMWASEVLLLLLLGSSRAVT  PGLDVSTAPGLDGSIPPGLDGSIPPGLDGSVSPGLDGSVSPGLDGSVSPGLDGSASPGLDGSTPAGRD

                                                                    Cathelin domain
G. gallus (fowlicidin-1)   (20) ---------------------------   LPAPLGYSQALAQAVDSYNQRPEVQNAFRLLSADPEPGPNVQLSSLHNLNFTIMETRCQARSGAQL
G. gallus (fowlicidin-2)   (20) ---------------------------   LPAPLSYPQALIQAVDSYNQRPEVQNAFRLLSADPEPGPGVDLSTLRALNFTIMETECTPSARLPV
G. gallus (fowlicidin-3)   (20) ---------------------------   LPAPLGYSQALAQAVDSYNQRPEVQNAFRLLSADPEPGPNVQLSSLHNLNFTIMETRCQARSGAQL
G. gallus (fowlicidin-B1)  (91) GTITPKLEGTITPKQDGSISPSWPWRW      PITYLDAILAAVRLLNQRISGPCILRLREAQPRPGWVGTLQRRREVSFLVEDGPCPPG----V

                                                                          Mature peptide
G. gallus (fowlicidin-1)   (84)  DSCEFKEDGLVKDCAAPVVLQGGRAVLDVTCVDSMADPV   RVKRVWPLV-----IRTVIAGYNLYRAIKKK-------
G. gallus (fowlicidin-2)   (84)  DDCDFKENGVIRDCSGPVSVLQDTPEINLRCRDASSDPV   LVQRGRFGRFLR-KIRRFRPKVTITIQGSARFG-----
G. gallus (fowlicidin-3)   (84)  DSCEFKEDGLVKDCAAPVVLQGGRAVLDVTCVDSMADPV   RVKRFWPLVPV--AINTVAAGINLYKAIRRK-------
G. gallus (fowlicidin-B1)  (177) -DCRSCEPGALQHCVGTVSIEQ-QPTAELRCRPLRPQ    PIRNWWIRIWEWLNGIRKRLRQRSPFYVRGHLNVTSTPQP
```

b

	Signal peptide			Cathelin domain			Mature peptide		
	Ka	*Ks*	*Ka/Ks*	*Ka*	*Ks*	*Ka/Ks*	*Ka*	*Ks*	*Ka/Ks*
**fowlicidin-1/fowlicidin-2**	0.028548	0.068385	0.417459	0.00	0.011512	0.00	1.10066	1.33253	0.825989
**fowlicidin-1/fowlicidin-3**	0.00	0.00	NA	0.00	0.012723	0.00	0.215999	0.558091	0.387032
**fowlicidin-1/fowlicidin-B1**	0.458366	0.292064	1.5694	0.00	0.00	NA	0.909783	0.534689	1.70152
**fowlicidin-2/fowlicidin-3**	0.028548	0.068385	0.417459	0.00	0.00	NA	0.324671	0.637693	0.509134
**fowlicidin-2/fowlicidin-B1**	0.515432	0.292064	1.76479	0.547835	0.7012463	0.781231	0.872453	0.508732	1.714956
**fowlicidin-3/fowlicidin-B1**	0.458366	0.292064	1.5694	0.656431	0.797205	0.823416	0.00	0.00	NA

Table S3 │ Evidence for adaptive evolution of the avian CATH-2 family. a, multiple sequence alignment of the precursors of avian CATH2s. Conserved residues are shaded. b, nonsynonymous substitution rate *Ka*, synonymous substitution rate *Ks*, and the ratio between them. "—" represents incomplete sequences; NA , not applicable ($Ks = 0$).

a

Species	Signal peptide	Cathelin domain	
C. coturnix (Cc-CATH2)	MLSCWVLVLALLGGVCA	LPAP--LSYPQALIQAVDTYNQRPEAQNAFRLLSADPEPGPGIDLNTLRELNFTIMETECVPSARTRIDDCDF	88
G. gallus (fowlicidin-2)	MLSCWVLLLALLGGVCA	LPAP--LSYPQALIQAVDSYNQRPEVQNAFRLLSADPEPGPGVDLSTLRALNFTIMETECTPSARLPVDDCDF	88
M. gallopavo (Mg-CATH2)	MASCWVLVLALLGGVCA	LPAP--LSYPQALTQAVDSYNQRPEVQNAFRLLSADPEPGPGVDLSTLRALNFTIMETECLPRAPMPINDCDF	88
P. colchicu (Pc-CATH2)	MLSCWVLVLALLGGVCA	LPAP--LSYPQALTQAVDSYNQRPELQNAFRLLSADPEPGPGVDLSTLRTLNFTIMETECVPRAQTPIDDCDF	88
M. undulates (Mu-CATH2)	MPSSWALVLVVLGGACA	LPAPAPLAYTQALAQAVESYNQRPEVQNSFRLLSADPEPAPGVDLTMLRVLNFSMMETECSPSSRMNPEDCDF	90
C. livia (Cl-CATH2)	MAGCWVLVLALLGGACA	LPAP--LAYTQALAQAVDSYNQRPEVPNAFRLLSADPEPAPGVELSSLRLLNFTMMETECTPSARVNPDDCDF	88
F. cherrug (Fc-CATH2)	MLSSWVLVLVVLGGACA	LPAPSPLAYTQALAQAVDSYNQRPEVQNVFRLLSADPEPAPGVELSVLRGLNFTIMETECTAGTRVNPDDCDF	90
F. peregrinus (Fp-CATH2)	MLSSWVLVLVMLGGACA	LPAPFPLAYTQALAQAVDSYNQRPEVQNVFRLLSADPEPAPGVELSVLRGLNFTIMETECTAGTRVNPDDCDF	90
P. humilis (Ph-CATH2)	MASSWVLLLAVLGGACA	LPAPAPLAYIQVLSQAVDSYNQRPEVKNAFRLLSAEPEPAPGVELSSLRGLNFSLMETDCAASARISPDDCDF	90
A. platyrhynchos (Ap-CATH2)	-----------------	------------LAQAVDSFNQRPEVHNAFRLLSADPEPAPGIDLGTLRALNFTFGLPVLG-----S------	50

Species		Mature peptide	
C. coturnix (Cc-CATH2)	KENGAIKDCSGPVTILQDAPEISLNCRDASSDPV	LVQRGRFGRFLKKVRRFIPKVIIAAQIGSRFG--	154
G. gallus (fowlicidin-2)	KENGVIRDCSGPVSVLQDTPEINLRCRDASSDPV	LVQRGRFGRFLRKIRRFRPKVTITIQGSARFG--	154
M. gallopavo (Mg-CATH2)	KENGVIRDCSGPVTILQDTPEINLRCRDASSDPV	LVQRGRFGRFLSKFRRFRPRVTITIQGSARFG--	154
P. colchicu (Pc-CATH2)	KENGVIRDCSGPVTILQDTPEINLRCRDASSDPV	LVQRGRFGRFLSKIRRFRPKFTITIQGSGRFG--	154
M. undulates (Mu-CATH2)	KENGVIRECSGPVQFLQTAPEIDLNCNDASSNPV	LVQRGRFGRFLGKIRRFRPKIRISGRADLTIRVG	158
C. livia (Cl-CATH2)	KENGVIKECSGPVQFGQSSPEIDLHCTDASSDPV	LIQRGRFGRFLGRIRRFRPRINFDIRARGSIRLG	156
F. cherrug (Fc-CATH2)	KEHGVIKECSGPVQFLQSSPEIDLRCSDASSNPV	LIQRGRFGRFLSRIRHFRPRISFDVRARGSIRLG	158
F. peregrinus (Fp-CATH2)	KEHGVIKECSGPVQFLQSSPEIDLHCSDASSNPV	LIQRGRFGRFLSRIRRFRPRISFDVHARGSIHLG	158
P. humilis (Ph-CATH2)	KENGAIRECSGTVQLLQGSPEINLSCFDASSDPV	LIQRGRFGRFLGKIRHFRPRVKFDIRLKGSVGLG	158
A. platyrhynchos (Ap-CATH2)	-------QFWGPADTRP-SP--------LSPQPV	LVQRGRFGRFLGKIRHLRPRVRIRVKADATVSFG	102

b

	Signal peptide			Cathelin domain			Mature peptide		
	Ka	*Ks*	*Ka/Ks*	*Ka*	*Ks*	*Ka/Ks*	*Ka*	*Ks*	*Ka/Ks*
**Cc-CATH2/fowlicidin-2**	0.0284098	0.00	NA	0.0867364	0.310537	0.279311	0.196957	0.32223	0.611232
**Cc-CATH2/Mg-CATH2**	0.0571742	0.338924	0.168693	0.083352	0.326259	0.255478	0.232424	0.222693	1.0437
**Cc-CATH2/Pc-CATH2**	0.00	0.235572	0.00	0.0762522	0.356529	0.213874	0.252339	0.125309	2.01373
**Cc-CATH2/Mu-CATH2**	0.187953	0.147785	1.27181	0.185976	0.488405	0.380782	0.417395	0.668322	0.624541
**Cc-CATH2/Cl-CATH2**	0.137285	0.106335	1.29106	0.193873	0.469558	0.412884	0.416902	0.504998	0.825551
**Cc-CATH2/Fc-CATH2**	0.120547	0.00	NA	0.20256	0.431017	0.469959	0.453652	0.407365	1.11362
**Cc-CATH2/Fp-CATH2**	0.118724	0.00	NA	0.196672	0.43361	0.453569	0.450669	0.414519	1.08721
**Cc-CATH2/Ph-CATH2**	0.207087	0.277718	0.745675	0.190987	0.460794	0.414475	0.459484	0.807488	0.569028
**Cc-CATH2/Ap-CATH2**		—			—		0.367676	0.581576	0.632206
**fowlicidin-2/Mg-CATH2**	0.0879212	0.334015	0.263225	0.0441832	0.123672	0.35726	0.0442085	0.221011	0.200029
**fowlicidin-2/Pc-CATH2**	0.0283133	0.232434	0.121812	0.0533513	0.185829	0.287098	0.0442387	0.124424	0.355548
**fowlicidin-2/Mu-CATH2**	0.225661	0.145951	1.54614	0.166658	0.471372	0.353558	0.361937	0.604008	0.599225
**fowlicidin-2/Cl-CATH2**	0.172452	0.105067	1.64136	0.149479	0.423119	0.353279	0.319077	0.588587	0.542106
**fowlicidin-2/Fc-CATH2**	0.154785	0.00	NA	0.150625	0.439121	0.343014	0.334137	0.54791	0.60984
**fowlicidin-2/Fp-CATH2**	0.152379	0.00	NA	0.15563	0.441727	0.352322	0.332116	0.558536	0.594619
**fowlicidin-2/Ph-CATH2**	0.172176	0.273992	0.628398	0.171487	0.468757	0.365833	0.276429	0.516177	0.535532
**fowlicidin-2/Ap-CATH2**		—			—		0.53838	0.474546	1.13452
**Mg-CATH2/Pc-CATH2**	0.0569755	0.241158	0.236258	0.0261429	0.10958	0.238574	0.0589088	0.0831987	0.708049
**Mg-CATH2/Mu-CATH2**	0.186251	0.340358	0.54722	0.173342	0.44349	0.39086	0.368438	0.778459	0.473292
**Mg-CATH2/Cl-CATH2**	0.0725331	0.28318	0.256138	0.169359	0.430661	0.393253	0.348307	0.65882	0.528684
**Mg-CATH2/Fc-CATH2**	0.187244	0.335423	0.558232	0.166994	0.439204	0.38022	0.329596	0.420163	0.784446
**Mg-CATH2/Fp-CATH2**	0.184296	0.350684	0.525532	0.172075	0.441827	0.389462	0.327636	0.427763	0.765931
**Mg-CATH2/Ph-CATH2**	0.135872	0.513632	0.264531	0.189115	0.489499	0.386343	0.326004	0.667064	0.488715
**Mg-CATH2/Ap-CATH2**		—			—		1.49054	0.00	NA
**Pc-CATH2/Mu-CATH2**	0.187244	0.449408	0.416646	0.186857	0.531039	0.351871	0.349482	0.660158	0.529391
**Pc-CATH2/Cl-CATH2**	0.136783	0.382785	0.357336	0.178369	0.510452	0.349434	0.286236	0.483506	0.592001
**Pc-CATH2/Fc-CATH2**	0.120112	0.233346	0.514739	0.177796	0.511067	0.347893	0.290573	0.342961	0.847248
**Pc-CATH2/Fp-CATH2**	0.118297	0.243069	0.486679	0.182936	0.514286	0.355708	0.288889	0.348821	0.828188
**Pc-CATH2/Ph-CATH2**	0.206295	0.505297	0.408265	0.00	2.44947	0.00	0.242723	0.577411	0.420365
**Pc-CATH2/Ap-CATH2**		—			—		0.351162	0.488603	0.718707
**Mu-CATH2/Cl-CATH2**	0.207087	0.106716	1.94055	0.0993471	0.347179	0.286155	0.298971	0.461857	0.647324
**Mu-CATH2/Fc-CATH2**	0.0577705	0.146487	0.394373	0.101611	0.185864	0.546699	0.299367	0.344957	0.867839
**Mu-CATH2/Fp-CATH2**	0.0871128	0.152151	0.57254	0.101305	0.187708	0.539695	0.328225	0.321778	1.02004
**Mu-CATH2/Ph-CATH2**	0.120365	0.328705	0.36618	0.149778	0.333824	0.448674	0.368816	0.275376	1.33932
**Mu-CATH2/Ap-CATH2**		—			—		1.26042	0.00	NA
**Cl-CATH2/Fc-CATH2**	0.208218	0.105443	1.97471	0.0747086	0.302738	0.246777	0.0866608	0.295165	0.293601
**Cl-CATH2/Fp-CATH2**	0.204861	0.109357	1.87333	0.0699031	0.30439	0.22965	0.101632	0.299458	0.339386
**Cl-CATH2/Ph-CATH2**	0.154785	0.228173	0.678366	0.134571	0.320986	0.419244	0.151649	0.397538	0.38147
**Cl-CATH2/Ap-CATH2**		—			—		1.53719	0.00	NA
**Fc-CATH2/Fp-CATH2**	0.0280606	0.00	NA	0.0083567	0.00	NA	0.0411561	0.00	NA
**Fc-CATH2/Ph-CATH2**	0.120981	0.324135	0.373243	0.136115	0.304129	0.447557	0.208692	0.279052	0.747861
**Ap-CATH2/Fc-CATH2**		—			—		1.45526	0.00	NA
**Fp-CATH2/Ph-CATH2**	0.169212	0.28664	0.590332	0.147352	0.301064	0.489438	0.272332	0.257176	1.05893
**Fp-CATH2/Ap-CATH2**		—			—		1.45526	0.00	NA
**Ph-CATH2/Ap-CATH2**		—			—		1.15151	0.00	NA
